# Developmental Origins of Pregnancy Loss in the Adult Female Common Marmoset Monkey (*Callithrix jacchus*)

**DOI:** 10.1371/journal.pone.0096845

**Published:** 2014-05-28

**Authors:** Julienne N. Rutherford, Victoria A. deMartelly, Donna G. Layne Colon, Corinna N. Ross, Suzette D. Tardif

**Affiliations:** 1 Department of Women, Children, and Family Health, College of Nursing, University of Illinois at Chicago, Chicago, Illinois, United States of America; 2 Department of Anthropology, University of Illinois at Chicago, Chicago, Illinois, United States of America; 3 Rollins School of Public Health, Emory University, Atlanta, Georgia, United States of America; 4 Southwest National Primate Research Center/Texas Biomedical Research Institute, San Antonio, Texas, United States of America; 5 Texas A & M University - San Antonio, San Antonio, Texas, United States of America; 6 Department of Cellular and Structural Biology, Barshop Institute for Longevity & Aging Studies, University of Texas Health Science Center at San Antonio, San Antonio, Texas, United States of America; Xavier Bichat Medical School, INSERM-CNRS - Université Paris Diderot, France

## Abstract

**Background:**

The impact of the intrauterine environment on the developmental programming of adult female reproductive success is still poorly understood and potentially underestimated. Litter size variation in a nonhuman primate, the common marmoset monkey (*Callithrix jacchus*), allows us to model the effects of varying intrauterine environments (e.g. nutrient restriction, exposure to male womb-mates) on the risk of losing fetuses in adulthood. Our previous work has characterized the fetuses of triplet pregnancies as experiencing intrauterine nutritional restriction.

**Methodology/Principal Findings:**

We used over a decade of demographic data from the Southwest National Primate Research Center common marmoset colony. We evaluated differences between twin and triplet females in the number of pregnancies they produce and the proportion of those pregnancies that ended in fetal loss. We found that triplet females produced the same number of total offspring as twin females, but lost offspring during pregnancy at a significantly higher rate than did twins (38% vs. 13%, p = 0.02). Regardless of their own birth weight or the sex ratio of the litter the experienced as fetuses, triplet females lost more fetuses than did twins. Females with a male littermate experienced a significant increase in the proportion of stillbirths.

**Conclusions/Significance:**

These striking findings anchor pregnancy loss in the mother’s own fetal environment and development, underscoring a "Womb to Womb" view of the lifecourse and the intergenerational consequences of development. This has important translational implications for understanding the large proportion of human stillbirths that are unexplained. Our findings provide strong evidence that a full understanding of mammalian life history and reproductive biology requires a developmental foundation.

## Introduction

Developmental programming theory suggests that the intrauterine environment – the intersection of maternal ecology and fetal development [1] – can have a lasting impact on adult health and function. A consistent link has been found in humans and animal models between maternal energy status during gestation or low birth weight (a proxy of a stressed developmental milieu), and obesity, diabetes, chronic cardiovascular disease, and reduced immunocompetence in adolescent and adult offspring [2]–[4]. Birth weight is the most common proxy measure of the quality of the intrauterine environment in mammals; it is easily measured and is strongly linked to postnatal and adult outcomes in a wide variety of species, including humans [2], [5]. However, birth weight itself is a product of dynamic processes experienced by the mother prior to conception and by both mother and fetus throughout gestation, and cannot be taken to reflect the entirety of these intrauterine processes [1]. While low birth weight has associations with later life outcomes, the intrauterine environment may be altering development of physiological function in ways that are not reflected by birth weight. Developmental programming occurs across the range of birth weights, not just at the low end [6], [7]. For example, maternal dietary composition may have differential impact on fetal endocrine pancreas development leading to diabetes in later life, without producing reductions in fetal body weight [8].

Fetal number is another source of variation in the quality of the intrauterine environment that may have intergenerational effects. One of the classic life history tradeoffs is the balance between number and quality of offspring [9], [10]. As the number of offspring increases, individual weights decrease, sometimes with impact on mortality risk [10]. Increased fetal number is associated with reduced birth weight and greater perinatal mortality in many taxa (sheep [11]; wood rats [12]; red squirrels [13]; common marmosets [14], [15]; humans [16]). Litter size does not account entirely for variation in birth weight [17], suggesting that these phenomena and their downstream effects may be decoupled under certain circumstances. Little is known about the long-term life history and reproductive impact of litter size at birth when controlled for birth weight.

The common marmoset monkey (*Callithrix jacchus*), like all marmosets and tamarins (Order, Primates; Suborder, Anthropoidea; Family, Cebidae; Subfamily, Callitrichinae [18]), expresses a highly plastic reproductive phenotype, regularly producing litters ranging from one to five multizygotic fetuses in captivity. Twins and triplets are the most common litter sizes [19] and mixed sex litters occur frequently. This variability is tied to maternal ecology; elevated maternal mass is the best predictor of greater ovulation number and litter size [20]. Individual repeatability of litter size is low, and the litter size a female experiences as a fetus does not predict the litter size she will produce as an adult, together suggesting that litter size is not genetically constrained but ecologically responsive [16], [20], [21]. Importantly, several occurrences of triplets have been observed in wild callitrichine species as well (cotton-top tamarins [22]; common marmosets [23]; golden lion tamarins [24]). This suggests that conceiving and gestating (although not rearing [19], [25]) more than two fetuses may be a common a feature of callitrichine reproductive biology both in the wild and in captivity. Variation in fetal number presents the opportunity to model varying intrauterine environments and their long-term effects.

We have shown previously that triplet marmosets experience an intrauterine environment that is qualitatively poorer than that experienced by twins, based on differences in maternal: neonatal weight ratios and placental efficiency [1], [26] and microscopic characteristics of the placental interface [27], [28]. Further, while both twins and triplet marmosets born at high birth weights tend to grow into high-weight adults, low birth weight triplets are much more likely to grow into large adults than are low birth weight twins [29], [30]. This pattern of “centile crossing” over the lifecourse has been implicated in the developmental programming literature as the phenotype carrying the greatest risk of adult disease [31]–[34]. For these reasons, twin marmosets can be viewed as the “control” developmental phenotype, with triplets exhibiting the “restricted” developmental phenotype. Triplet females also carry the potential burden of greater exposure to prenatal androgens from their male littermates.

This paper first characterizes reproductive parameters in a colony of captive common marmoset monkeys (*Callithrix jacchus*) overall and according to litter size and birth weight, and then explores the relationship between a female marmoset’s birth condition (her litter size, intralitter sex ratio, and birth weight) and her risk of pregnancy loss in adulthood.

## Methods

### Ethics Statement

All animal procedures, husbandry, and housing were conducted according to Southwest National Primate Research Center Institutional Animal Care and Use Committee requirements.

### Colony and Housing

Demographic records from the Southwest National Primate Research Center in San Antonio, Texas dating from 1994 to 2012 were available for a total of 1395 animals of both sexes and all birth conditions. Because the intent was to focus on the intrauterine contribution to life history and reproductive output, analyses were restricted to females for whom a full complement of birth condition (weight and litter size) and adult reproductive parameters were known. Analyses were conducted on subsets of this group (i.e. twins and triplets). Adult females were housed with at least their adult male mate, but often with older offspring. It is not uncommon for the family group to contain adolescent, juvenile, and infant offspring at the same time. Family structure at the time of each pregnancy studied was not recorded and thus could not be considered in this study. Groups were housed according to Institutional Animal Care and Use Committee standards for marmosets.

### Coding Pregnancy Loss

In the original database birth status was coded as follows: STILL, meaning fully developed fetus delivered at term with no sign of earlier death *in utero* and/or no lung flotation; DIU (“dead *in utero*”), meaning a mostly or fully developed fetus either aborted or discovered at term delivery but showing clear evidence of *in utero* death preceding labor (macerated flesh, skin slippage, “mushiness”); and ABORT, meaning found or delivered before due date (gestation in common marmosets is ∼143 days, [35]). All of these categories were combined to generate total loss. Thus, early loss is likely conflated with the equivalent of antepartum stillbirth, i.e. fetal loss occurring in the last trimester but prior to labor and delivery. Related to this limitation, we do not have any record of offspring that were lost so early that there was no visible evidence of loss so we cannot extrapolate our findings to very early pregnancy loss. Further, given that placentophagy and even fetophagy are not uncommon practices and that parturition is typically nocturnal, our estimates of loss of even late gestation fetuses may be underestimated. Finally, we did not regularly conduct lung flotation, a highly accurate method of determining stillbirth in humans [36].

### Predictor and Outcome Variables

Litter size at birth was the most consistently used predictor variable. We evaluated the impact of litter size on total number of offspring produced, total litters produced, total lost fetuses, total affected litters (litters in which at least one fetus was lost), and total lost litters (litters wherein all fetuses were lost), all fitted as continuous variables. We controlled all regression models for birth weight, birth year (to control cohort effects), and early adult weight. As described by Tardif and Bales [29], early adult weight was measured between 17–22 months. This is a few months later than the average age of puberty (11–13 months) and precedes the average age of first conception (2.49 years) [19], but reflects the achievement of adult weight [37], [38].

### Statistics

Although litter size ranges from 1–5 in the captive common marmoset, analyses were restricted to twins and triplets because these are by far the two most representative litter sizes of origin of those females who survived to the age of maturity. All analyses were conducted using Stata for Windows, version 10 IC (StataCorp, College Station, TX). Two-tailed T-tests were used to compare twins and triplets to each other in terms of birth condition and other life history characteristics. Z-tests were used to test the significance of the difference in proportion of fetal losses between twin and triplet females. There were nine pairs of females (total n = 18 out of 62) in the sample who were born into the same litter. We used two-tailed T-tests to compare females with and without littermates in the study in terms of birth condition and the other life history characteristics.

In some cases we wanted to evaluate the predictive relationships between litter size and pregnancy loss variables; therefore, we used simple and multiple linear regression modeling. We assigned all females a litter ID (to control for those females born into the same litters as described above) and ran that ID as a random effect in regression models. We assumed that many of our predictor and outcome variables in these models would exhibit collinearity. Therefore, all models were evaluated for collinearity using the *estat VIF* command in Stata to measure the variance inflation factor. Models returning a VIF of >5 (indicative of high collinearity) were subject to rejection; none of our models returned a rejectable VIF.

### Data Availability

Raw data are stored in databases at the SNPRC and the University of Illinois at Chicago. Requests for data can be directed to the primary author.

## Results

### Birth Condition and Reproductive Demographics of the Southwest National Primate Research Center Female Marmosets

At the time of these analyses, there were 1395 animals in the Southwest National Primate Research Center marmoset colony database; a large proportion of this number includes animals that died before juvenility. Of adult animals, 113 were reproducing females. Not all females were born in the colony or entered the colony with birth data, so litter size at birth was known for 79 of these females, of which 75 were either twins (n = 37) or triplets (n = 38). These 75 females accounted for 94.95% of the reproducing females of known litter size at birth; remaining analyses are thus restricted to these twin and triplet females. When restricted to twin and triplet females of known birth weight and early adult weight, the sample size for analysis was 62 (twins = 30, triplets = 32; Table 1). Sex composition of a female’s birth litter was known for 27 twins and 29 triplets.

**Table 1:** Sample characteristics, stratified by litter size.

	All (n = 62)Mean (±SD)	Twins*(n = 30)Mean (±SD)	Triplets*(n = 32)Mean (±SD)	*P* value
Number of male littermates	0.84 (0.71)	0.48 (0.51)	1.22 (0.70)	**<0.00001**
**Birth weight (bw), g**	29.90 (3.19)	31.17 (3.29)	28.72 (2.62)	**0.002**
Early adult weight (eadwt), g	414.09 (83.07)	403.76 (75.87)	423.78 (89.41)	0.35
**Low bw & high eadwt** ** **, ^@^**	28%	13.79%	40.74%	**0.01**
Age at first reproduction, years	2.94 (0.62)	3.00 (0.11)	2.88 (0.11)	0.43
Total number of litters	3.92 (3.28)	3.87 (2.67)	3.97 (3.80)	0.90
Triplet litters, out of total litters	40.58%	47.63%	33.97%	0.14
Total number of offspring	9.81 (8.68)	9.90 (7.31)	9.72 (9.92)	0.97
**% Offspring lost** **	26.03%	12.97%	38.27%	**0.02**
Affected litters***, out of total litters**	35.85%	26.70%	44.42%	0.14
Entire litter lost, out of total litters**	22.15%	12.55%	31.15%	0.07

@ Median split: low birth weight ≤27.86 g, high adult weight ≥479.20.

*Unpaired two-tailed T-test.

**Out of total number of offspring; Difference in proportion, unpaired two-tailed Z-test.

***Litter affected by loss of at least one fetus.

Females who had littermates in the study (n = 20) did not differ from the rest of the sample (n = 42) in litter size, birth weight, or adult weight; they had significantly fewer male littermates than the rest of the sample (Table S1). Triplets were born at significantly lower birth weights than twins, but did not differ significantly in weight at the early adulthood mark (Table 1). Birth weight and adult weight were divided into high and low categories via median splits. Low birth weight triplets were significantly more likely to grow into high weight adults than were low birth weight twins.

### Impact of Birth Weight and Litter Size on Reproductive Parameters in Adulthood

Twins and triplets did not differ in their age at first reproduction, nor did they differ in the number of litters produced or the total number of offspring gestated (Table 1). Triplet females were not more likely than twin females to produce triplet litters. None of these outcomes differed when females were stratified on birth weight (Table S2). Females with littermates in the study did not differ from the rest of the sample in age at first reproduction or in the total number of offspring produced (Table S1).

**Figure 1:**
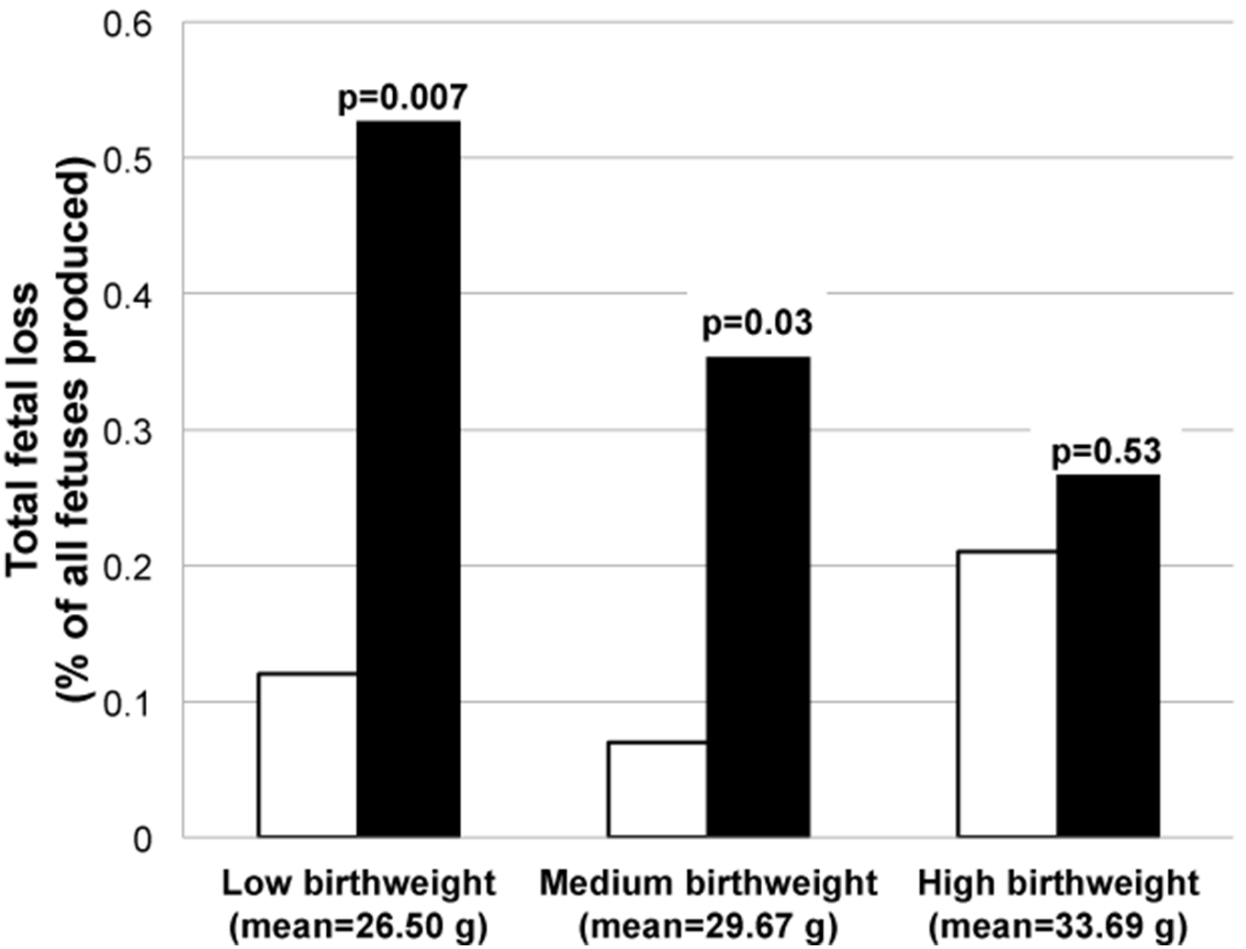
Pregnancy loss in twin and triplet adult females across three tertiles of the females’ own birth weights.

Despite the lack of difference in total offspring gestated, triplets lost three times as many offspring during pregnancy (Table 1). Triplet females tended to experience these losses across more pregnancies than did twins, with triplets losing entire litters 2.48 times more than twins, though this difference was not significant (p = 0.07; Table 1). Similar analyses of adult females stratified by birth weight showed no differences in rates of loss (Table S2). Triplet females lost more fetuses in each of three categories of birth weight (Figure 1), with the difference being significant in low and medium weight categories. Triplet females who were born in the lowest birth weight tertile experienced the highest proportion of fetal loss in adulthood.

A multiple regression model including a female’s own litter size, birth weight, early adult weight, and birth year significantly predicted her rate of total loss, explaining 19% of the variance (Table 2: model 1). In this model, litter size was the sole significant independent predictor of a female’s rate of total stillbirth rate: the larger the litter at birth, the greater the rate of loss in adulthood. A multiple regression model excluding birth weight remained predictive of total loss, with litter size being the sole predictor of loss (Table 2: model 2). When litter size was excluded, none of the models were significant overall (Table 2: model 3). Models containing litter size tended to explain a greater proportion of the variance in the outcomes than those without. Litter size alone explained 18% of the variance in total loss, compared to only 7% for birth weight alone (Table 3: models 1 and 2).

**Table 2:** Multiple regression models predicting pregnancy loss in adult female marmosets (n = 62).

	Model 1: All predictor variablesβ (95% C.I.) Total*	Model 2: Birth weight excludedβ (95% C.I.) Total**	Model 3: Litter size excludedβ (95% C.I.) Total
Litter size at birth	0.23** (0.07, 0.39)	0.26 *** (0.11, 0.40)	–
Birth weight (g)	−0.01 (−0.04, 0.02)	–	−0.03* (−0.05, −0.003)
Birth year	0.0007 (−0.02, 0.02)	−0.001 (−0.02, 0.02)	0.004 (−0.02, 0.02)
Adult weight (g)	−0.0002 (−0.001, 0.001)	−0.0003 (−0.001, 0.001)	0.0001 (−0.001, 0.001)
Model R^2^	0.19	0.18	0.08

****p< = 0.0001,

***p< = 0.001,

**p< = 0.01,

*p< = 0.05.

**Table 3:** Simple regression models predicting pregnancy loss in adult female marmosets (n = 62).

	Model 1: Birth weight only β (95% C.I.)	Model 2: Litter size only β (95% C.I.)
Litter size at birth	–	0.25*** (0.11, 0.39)
Birth weight (g)	−0.03* (−0.05, 0.002)	–
Model R^2^	0.07	0.18

****p< = 0.0001,

***p< = 0.001,

**p< = 0.01,

*p< = 0.05.

**Figure 2:**
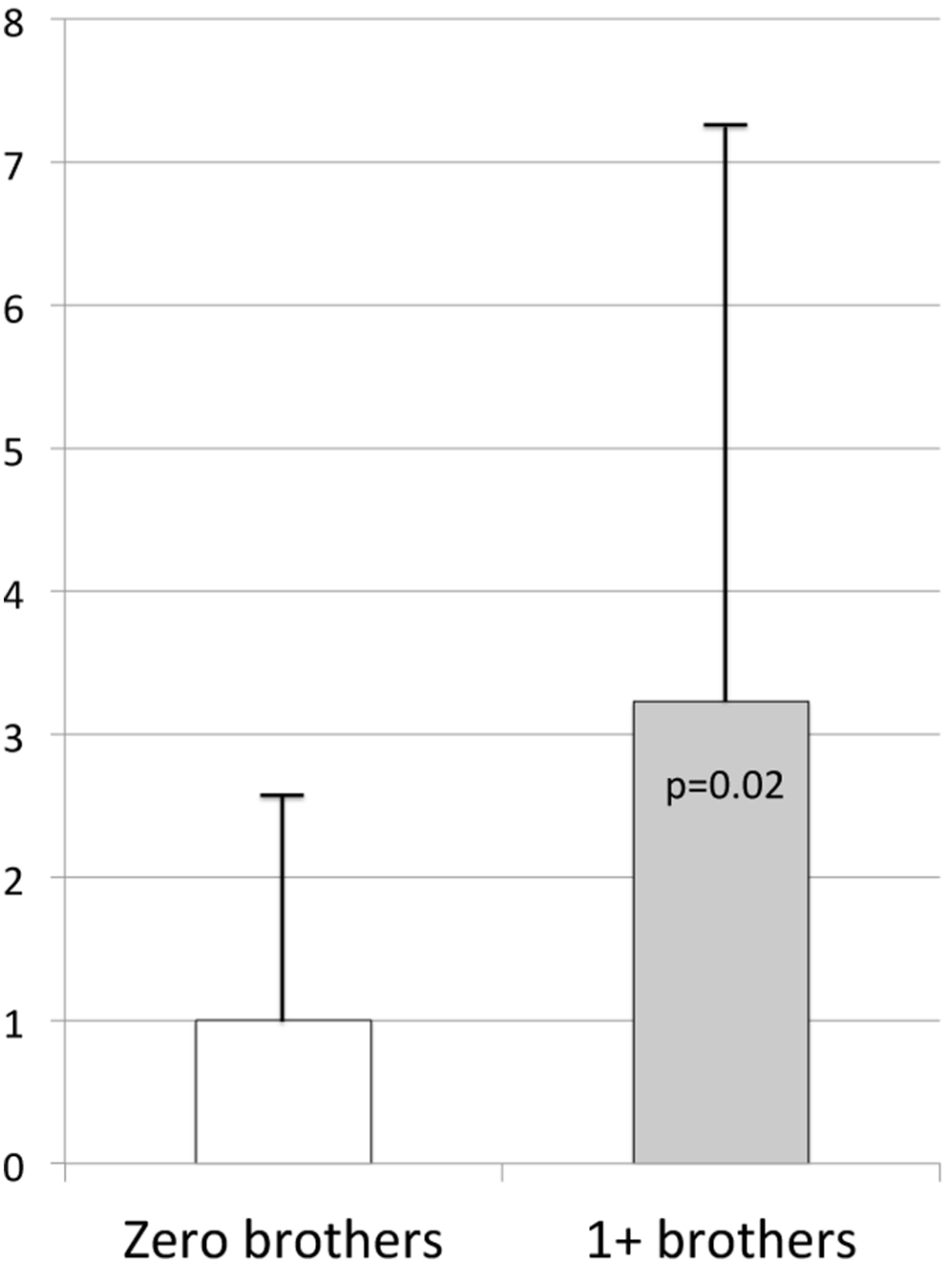
Pregnancy loss across all adult females according to number of brothers with whom they shared the womb during their own fetal period.

**Figure 3:**
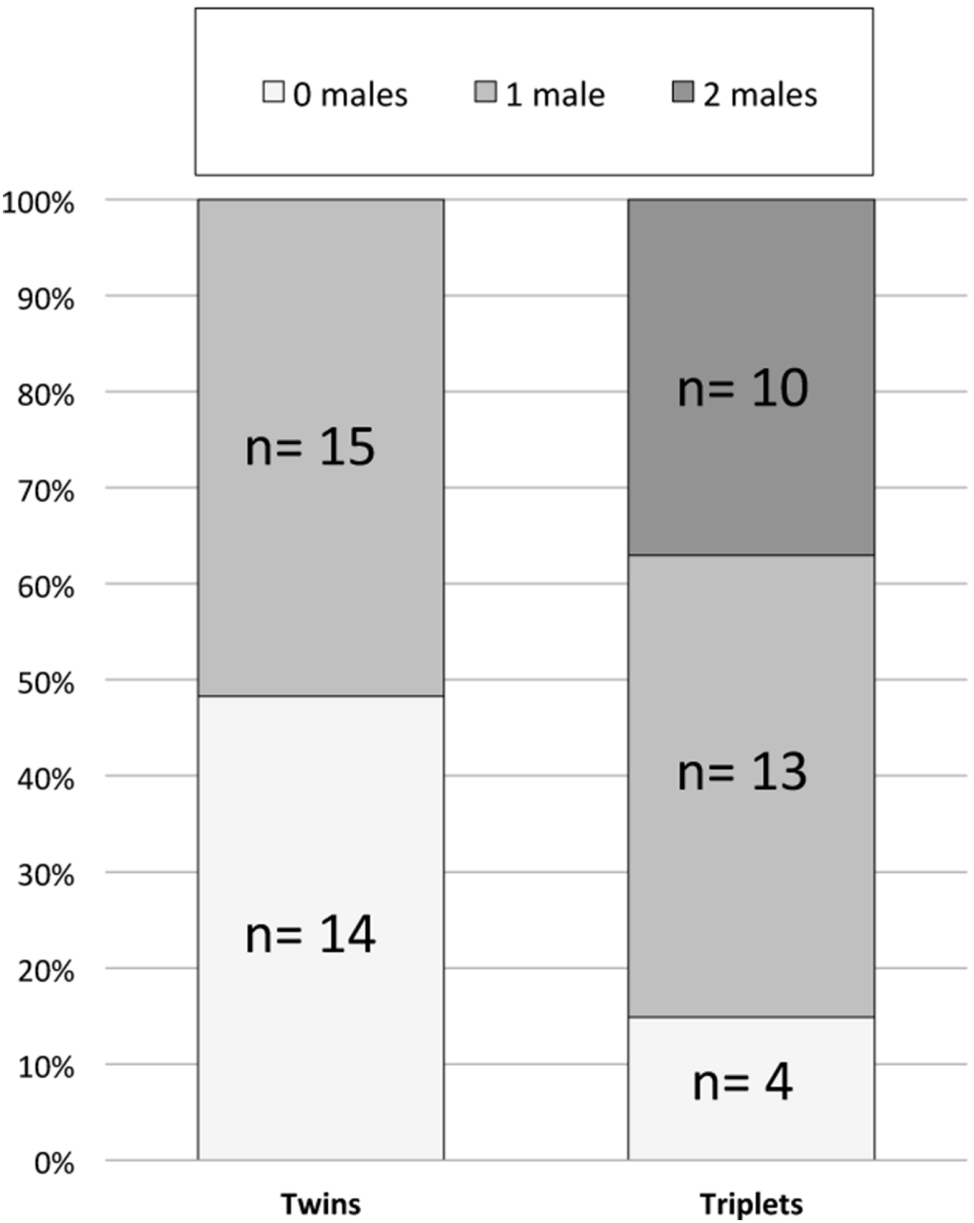
Distribution of brothers with whom twin and triplet females shared the womb during their own fetal period.

The impact of exposure to male littermates on pregnancy success in the adult females was assessed in three ways. First, females with littermates in the study differed from the rest of the sample in being less likely to have had a male littermate compared to the rest of the sample. These females did not differ in the number of total offspring produced, but they had significantly lower loss rates compared to the rest of the sample (Table S1). Second, females with one or more brother regardless of litter size experienced a significant increase in fetal loss compared to females from female-only litters (Figure 2). Third, male exposure as a function of litter size was assessed. Twin females had an average of 0.48 brothers *in utero*, compared to triplets who had 1.22 brothers (p<0.00001; Table 1). Twin females were equally likely to have had either a male or a female littermate (Figure 3). In contrast, 85% of triplet females had either one or two male littermates; only 4 of the 27 triplet females for whom birth sex ratio was known were born into all-female litters (Figure 3). The triplets did not exhibit a significant dose response of fetal loss to having one versus two male littermates (z = 0.33, p = 0.74; data not shown). A categorical variable (zero versus one or more) was thus constructed for both twins and triplets. Twin females lost 2.91 more fetuses when they were exposed to a brother (p = 0.05, Table 4) as opposed to a sister. There was not a significant effect of male exposure on fetal loss in the triplet females.

**Table 4:** Number of fetuses lost by females who had zero vs. one or more male littermates (unpaired T-test).

	Twin females (n = 29)					Triplet females (n = 27)				
	N	Mean (S.D.)	*t*	df	p	N	Mean (S.D.)	*t*	df	p
0 male littermate	15	0.87 (1.36)	−2.08	27	0.05	4	1.50 (2.38)	−0.96	25	0.34
1+ male littermate	14	2.53 (3.15)				23	3.70 (4.38)			

## Discussion

Consistent with our previous work, triplets were born at significantly lower birth weights than were twins. Further, triplets were significantly more likely than twins to exhibit a lower birth weight/higher adult weight profile. This is consistent with the classic growth-restricted phenotype that is typically thought to present the highest risk within the developmental programming paradigm [30], strengthening the concept of the marmoset monkey triplet as a model of disrupted growth and development. In further support, females born as triplets lose nearly three times as many offspring before birth than do females born as twins. This robust and highly striking finding speaks to the importance of considering a female’s own intrauterine development when considering adult reproductive success and thus fitness. Triplet marmosets are born at lower birth weights than twins raising the possibility that birth weight may be driving fetal loss in adulthood. However, analyses of the effect of birth weight separate from litter size do not support this conclusion. Litter size is independent of birth weight as a risk factor for pregnancy loss in adult female marmosets, strong evidence that differential developmental trajectories not reflected in birth weight have a critical impact on reproductive outcomes in adulthood. That litter size in the marmoset and other species is related to maternal energetic condition at multiple time points (e.g. ovulation, conception, gestation) suggests that dynamic and complex physiology-ecology interactions within and between generations are central to understanding the evolution of life history variation within and between species.

Maternal birth weight influences fertility [39], offspring birth weight, preterm delivery, infant and perinatal mortality [40]. However, only a few studies have demonstrated a link between proxies of a female’s own growth *in utero* and fetal loss experienced in adulthood. For example, women who as a fetus experienced nutrient restriction during the third trimester due to the Dutch Famine during World War II had a significantly higher rate of stillbirth and perinatal mortality than women who were not exposed to famine as fetuses [41]. In female rhesus macaques, low maternal birth weight was associated with producing more stillborn offspring as well as later age at first reproduction and smaller offspring [42]. The marmoset model presented here augments these previous findings while allowing us to view developmental programming across the range of birth weight. Triplets had poorer reproductive outcomes than twins across all categories of birth weights, even “normal” birth weight. Similar birth weights in two infants may or may not predict the same outcome [1]. Restricted or redistributed nutrient flow may not result in overall reductions in birth weight in order to maintain adequate brain and somatic growth, while non-essential systems (e.g. reproductive organs, hypothalamus-pituitary-ovarian (HPO) axis) may be affected adversely. Thus, average-sized marmoset triplets may have “growth-impaired” reproductive phenotypes, which would account for significant litter size differences in offspring viability regardless of birth weight. That said, reproductive function in triplet females appears to be particularly poor for those born at low weights.

The source of such extreme discrepancies in the triplet marmoset female’s ability to successfully gestate fetuses to term is unclear, though some lines of evidence suggest that differential development of the HPO axis and reproductive tract play important roles. For example, small for gestational age (SGA) adolescent girls have been reported to exhibit reduced ovarian dimensions [43] and reduced Follicle-Stimulating Hormone (FSH) at 18 years of age [44]. Adult reproductive function thus could have fetal origins in the development of the hypothalamus and pituitary, which in turn would have an impact on both pituitary FSH production and ovarian organogenesis. Even earlier on the developmental timeline, germ layer migration and differentiation could contribute to differential HPO function. These findings suggest the possibility that triplet marmosets experience altered intrauterine development affecting ovarian size, the quality of the primordial follicle pool, precedents of endometrial function (with implications for implantation and placentation), and even the HPO axis. Uterine size and vasculature may also be altered. Limiting the physical or functional capacity of the uterus in a litter-bearing primate could have direct effects on the ability to gestate live offspring to term. Prospective studies are underway to track the development and function of these systems from birth to first pregnancy in female marmosets in this colony.

Transfer of prenatal testicular androgens to female fetuses as a function of mixed sex litters is a key factor driving differential reproductive development in several mammalian species. For example, female mice flanked by brothers *in utero* have a longer anogenital distance (AGD) which is considered a masculinized phenotype [45]. In female swine, number of male littermates is associated with a longer AGD [46]. Some physiological correlates of male littermate androgen exposure in female mice include increased circulating testosterone at birth [47] and in adulthood, decreased likelihood to become pregnant [48] and fewer viable litters [49]. Data are sparse for similar effects in marmosets or their close litter-bearing relatives, the tamarins. Prenatal androgen levels in marmosets are variable and thought to be largely of maternal or placental origin, as they are apparently not related to overall litter size or presence or number of male fetuses [50], [51]. However, given current methods, it is still unknown to what extent male marmoset fetuses are producing testicular androgens [51]. Regardless of the source, marmosets are often described as escaping the virilizing effects of prenatal androgens, in part because much of genital differentiation occurs postnatally [52]. However, one colony reported a high incidence (∼32%) of ambiguous or masculinized genitalia in female marmoset newborns [53]. In recent years, there have been two reports of individual female marmosets or tamarins with masculinized genitalia that express either the testis-determining *Sry* gene, the Y-linked zinc finger protein gene (ZFY), or both [54], [55]. The ZFY female described in Smith et al. (2013) had a male littermate, making it “difficult to disentangle genetic from endocrine influences” (p.110, [52]). Given the current state of understanding, the organizing effects of male testicular androgens on their female siblings *in utero* cannot be ruled out. At this point it is also entirely unknown whether or the extent to which placental androgens differ in mixed sex marmoset litters.

The results of the current study further suggest that male exposure *in utero* may indeed have an effect on a female’s reproductive development in the marmoset monkey. Although we did not have direct measures of androgen levels, we observed that females who shared the womb with any brothers were significantly more likely to lose offspring during gestation, with loss rates threefold greater than those females from all female litters. In our sample, this effect is most apparent in twins. Twin females lost significantly more offspring in adulthood when they shared the womb with a brother instead of a sister. The existence of a “brother effect” on twin female marmosets is potential evidence for an organizing effect of testicular androgens. If this is the case, then it is reasonable to expect that triplets would exhibit a higher degree of loss with increasing potential exposure to brothers *in utero*. However, the effect on triplet females was not significant, regardless of the number of male littermates. Our sample size may be inadequate to tease out a “brother effect” in triplets. There were only four all-female triplet litters, compared to the even distribution of male-female and female-female twin litters, precluding appropriate statistical testing of differences.

The pattern thus far elucidated hints that disordered reproductive development leading to pregnancy loss in triplet marmoset females is severe. It is important to note that among mammals, primates are unique in having what is called the fetal zone of the adrenal gland, which actively produces the androgens dehydroepiandrosterone (DHEA), DHEA-sulfate (DHEA-S), and androstenedione during gestation [56]. The placenta aromatizes adrenal androgens to estrogen, thus providing a buffer to female fetuses [57]. However, this phenomenon is best studied in primates that produce singletons; little is known about this process in the litter-bearing marmosets and tamarins. Further, the pattern of placental corticotrophin releasing hormone (CRH) production differs between the monkeys and apes (including humans) [58]. CRH is the hormone – usually produced by the hypothalamus - that stimulates the pituitary to produce adrenocorticotrophin (ACTH). In turn, ACTH stimulates the fetal zone of the adrenal gland to produce androgens and glucocorticoids. In apes and humans, high levels of CRH are maintained throughout pregnancy and are correlated with estrogen levels. In contrast, in the marmoset monkey and the baboon CRH levels rise early in gestation, peak mid-gestation, and then drop precipitously, suggesting that human patterns in this regard may not be applicable to the marmoset [58]. Together, these findings raise the possibility that female fetuses may be more vulnerable to prenatal androgens in the litter-bearing marmosets and tamarins than in other primates. Thus, we speculate that the difference in baseline reproductive performance between twin and triplet marmoset females may be due at least in part to the cumulative effect of adrenal androgen production and insufficient placental buffering across more fetuses in triplet litters, which could then be exacerbated by testicular androgen production by brothers. Another explanation of the “brother effect” could be that males are born at larger birth weights, thus monopolizing maternal nutrients and disrupting female littermate development through nutrient allocation. However, males and females are born at similar birth weights. Certainly more comprehensive data are needed to fully interrogate the intrauterine impact of brothers versus sisters on female, specifically the role of cumulative levels of adrenal androgens and related placental function.

Developmental processes may underlie the large proportion of unexplained stillbirths in humans. Stillbirth is characterized as a multifactorial outcome with various risk factors contributing in large and small ways to an overall risk profile that varies widely across populations. Common maternal predictors of stillbirth in high-income countries include prepregnancy obesity, diabetes, chronic hypertension, infection, smoking, increasing maternal age, and lack of prenatal care [59]. In low-income countries, maternal infectious disease is the major identifiable risk factor [60]. Nearly 45% of all stillbirths are thought to be preventable through the modification of risk phenotypes (diabetes control, weight loss, smoking cessation, prenatal care, infection protection, etc.) [61]. Given that this leaves the majority of stillbirths unaccounted for, it is clear that not all risk factors have been identified. We argue that a consideration of the developmental experience of the mother may help close this gap. In our study, litter size alone explains 18% of the variance in stillbirths (p<0.001), indicating that considering birth history could add substantial power to current models of human stillbirth.

There are potential limitations of our study. First, though the marmoset is an anthropoid primate with many similarities to humans, the fact that it produces multiples as a matter of course differentiates its reproductive physiology from that of the human, typically a producer of singletons. The genetic mechanisms of litter size in marmosets are beginning to be elucidated, with potential for understanding multiple births of both natural and assisted origin in humans [62]. Since the etiology of multiple births is likely to differ between humans and marmosets, the marmoset model is possible better viewed as one of intrauterine nutrient restriction due to natural variation in litter size, as opposed to an analog for human multiples. Second, since this was a retrospective study of demographic records, the extant coding system did not clearly differentiate early pregnancy loss from *sensu stricto* stillbirth (the loss of a fetus at a developmental stage equivalent to 28 weeks of human gestation) so that losses span both the embryonic and fetal periods. In our planned prospective studies, specific temporal categories of loss will be employed. A third consideration is the impact of secular trends in birth weight, litter size, and adult weight on reproductive parameters. Individuals are being born at greater weights into larger litters and growing into larger adults than they were during the early years of the colony. The differential effects of litter size and birth weight reported here are independent of cohort effects, suggesting they are robust phenomena unaffected by secular trends; this strengthens the observation that developmental programming operates across a range of birth weights. Finally, our focus is on the influence of a female’s own early life characteristics on her adult reproductive function. The influence of the female’s mate was not considered in this study. Although paternal contributions such as age, sperm quality, and parental care are important to reproductive success and fetal outcomes, they were beyond the scope of the current study. Future analyses of such contributions and their impacts are planned.

In summary, our data overall clearly show that fetal development has a tremendous impact on adult reproductive function: triplets lost three times as many fetuses as did twins. A female common marmoset monkey’s own litter size at birth – a phenotype reflective of a nutritionally or otherwise stressed fetal environment – acts on her ability to successfully gestate fetuses to term, perhaps via the development of the HPO axis and reproductive tract. This may be due to a combination of changes in nutritional allocation and prenatal androgen exposure, both of which may alter developmental pathways. We suggest there are specific developmental mechanisms that entrain reproductive phenotypes and life history schedules across generations, providing a novel way of framing life history plasticity and evolution in litter-bearing mammals. While there are obvious applications of our work to life history studies of litter size and the physiology of multiple pregnancies, the broader implications of our marmoset model transcend these phenomena, situated in the ability to model a naturally-occurring “developmental programming” or “growth-impaired” phenotype (triplets) compared to a “normal” or “control” phenotype (twins). Our findings provide strong evidence that a full understanding of mammalian life history, reproductive biology, and pregnancy outcomes requires a developmental foundation.

## Supporting Information

Table S1 **Sample characteristics of females with and without littermates in the study.**
(DOCX)

Table S2 **Sample characteristics, stratified by birth weight.**
(DOCX)
