# Supplementary material for: Developmental Origins of Pregnancy Loss in the Adult Female Common Marmoset Monkey (Callithrix jacchus)
Source: PLoS One. 2014 May 28;9(5):e96845. doi: 10.1371/journal.pone.0096845 (PMC4037172; doi:10.1371/journal.pone.0096845)
Supplement: Table S1 — Sample characteristics of females with and without littermates in the study. (DOCX) [file pone.0096845.s001.docx]

Table S1: Sample characteristics, stratified by females with and without littermates in the study

|  | All (n=62)  Mean (±SD) | No littermates in study* (n=42)  Mean (±SD) | Littermates in study* (n=20)  Mean (±SD) | *P* value |
| --- | --- | --- | --- | --- |
| Litter size | 2.52 (0.50) | 2.52 (0.51) | 2.50 (0.51) | 0.86 |
| **Number of male littermates** | N=56: 0.84 (0.71) | N=38: 1.13 (0.62) | N=18: 0.22 (0.43) | **<0.00001** |
| Birth weight (bw), g | 29.90 (3.19) | 29.50 (3.29) | 30.75 (2.85) | 0.15 |
| Early adult weight, g | 414.09 (83.07) | 410.02 (87.38) | 422.65 (74.62) | 0.58 |
| Age at first reproduction, years | 2.94 (0.62) | 3.02 (0.60) | 2.77 (0.64) | 0.15 |
| Total number of litters | 3.92 (3.28) | 3.62 (2.64) | 4.55 (4.33) | 0.30 |
| Triplet litters, out of total litters** | 40.58% | 39.67% | 42.51% | 0.77 |
| Total number of offspring | 9.81 (8.68) | 8.98 (7.11) | 11.55 (11.33) | 0.28 |
| **% Offspring lost**** | 26.03% | 32.13% | 13.21% | **0.02** |
| Affected litters***, out of total litters** | 35.85% | 41.35% | 24.28% | 0.09 |
| Entire litter lost, out of total litters** | 22.15% | 26.35% | 13.32% | 0.10 |

* Unpaired two-tailed T-test

** Out of total number of offspring; Difference in proportion, unpaired two-tailed Z-test

***Litter affected by loss of at least one fetus
