# Supplementary material for: Developmental Origins of Pregnancy Loss in the Adult Female Common Marmoset Monkey (Callithrix jacchus)
Source: PLoS One. 2014 May 28;9(5):e96845. doi: 10.1371/journal.pone.0096845 (PMC4037172; doi:10.1371/journal.pone.0096845)
Supplement: Table S2 — Sample characteristics, stratified by birth weight. (DOCX) [file pone.0096845.s002.docx]

Table S2: Sample characteristics, stratified by birth weight (median split)

|  | All (n=62)  Mean (±SD) | Lower birth weight* (n=37)  Mean (±SD) | Higher birth weight* (n=25)  Mean (±SD) | *P* value |
| --- | --- | --- | --- | --- |
| **Litter size** | 2.52 (0.50) | 2.68 (0.47) | 2.28 (0.46) | **0.002** |
| **Number of male littermates^@^** | 0.84 (0.71) | 1.03 (0.75) | 0.60 (0.58) | **0.02** |
| **Birth weight (bw), g** | 29.90 (3.19) | 27.79 (2.01) | 33.09 (1.93) | **<0.00001** |
| Early adult weight, g | 414.09 (83.07) | 401.06 (82.27) | 433.38 (82.09) | 0.13 |
| Age at first reproduction, years | 2.94 (0.62) | 2.95 (0.11) | 2.92 (0.12) | 0.86 |
| Total number of litters | 3.92 (3.28) | 4.11 (3.75) | 3.64 (2.45) | 0.59 |
| Triplet litters, out of total litters | 40.58% | 35.51% | 48.09% | 0.18 |
| Total number of offspring | 9.81 (8.68) | 9.14 (9.37) | 9.60 (7.77) | 0.84 |
| % Offspring lost** | 26.03% | 29.44% | 20.98% | 0.45 |
| Affected litters***, out of total litters** | 35.85% | 37.97% | 32.71% | 0.68 |
| Entire litter lost, out of total litters** | 22.15% | 26.42% | 15.82% | 0.31 |

^@^ Sex ratio known for n=56: Lower birth weight, n=38; Higher birth weight, n=18

* Unpaired two-tailed T-test

** Out of total number of offspring; Difference in proportion, unpaired two-tailed Z-test

***Litter affected by loss of at least one fetus
